# Integrated Cervical Self-Sampling for Cytology, High-Risk Human Papillomavirus, and Sexually Transmitted Infection Testing: A Prospective Study

**DOI:** 10.3390/diagnostics16121863

**Published:** 2026-06-16

**Authors:** Chang Gok Woo, Jaehoon Choi, Yujin Im, Man Ki Kim, So Young Kim, Jong Hyock Park, Ok-Jun Lee

**Affiliations:** 1 Department of Pathology, College of Medicine, Chungbuk National University, Cheongju 28644, Republic of Korea; thewallflower@daum.net (C.G.W.); cndskach@naver.com (J.C.); yujinim99@naver.com (Y.I.); 2 Department of Pathology, Chungbuk National University Hospital, Cheongju 28644, Republic of Korea; 3 Motaean Woman Hospital, Cheongju 28628, Republic of Korea; windyu1@hanmail.net; 4 Department of Health Policy and Management, College of Medicine, Chungbuk National University, Cheongju 28644, Republic of Korea; letter.sykim@gmail.com; 5 Department of Public Health and Preventive Medicine, Chungbuk National University Hospital, Cheongju 28644, Republic of Korea; 6 Department of Preventive Medicine, College of Medicine, Chungbuk National University, Cheongju 28644, Republic of Korea; 7 Institutes of Health & Science Convergence, Chungbuk National University, Cheongju 28644, Republic of Korea

**Keywords:** cervical cancer screening, self-sampling, cytology, human papillomavirus, sexually transmitted infections, preventive medicine

## Abstract

**Background/Objectives:** Cervical self-sampling is increasingly used for high-risk human papillomavirus (hrHPV) testing, but evidence for integrated liquid-based cytology (LBC) and sexually transmitted infection (STI) testing is limited. This study evaluated the feasibility and diagnostic agreement of an integrated single-step self-sampling approach for LBC, hrHPV, and STI testing. **Methods:** In this prospective paired study, 520 Korean women for cervical cancer screening between December 2024 and February 2025 were enrolled. Each participant first underwent cervical self-sampling using Earlypap^®^, followed by clinician-collected sampling. Paired specimens were analyzed for LBC, hrHPV, and STI detection. Percentage agreement and Cohen’s kappa coefficients (κ) were calculated. **Results:** Self-sampling was successful on the first attempt in 98.5% of participants, with 92.1% preferring it over clinician-collection. LSIL was detected in 2.3% of self-collected and 1.2% of clinician-collected specimens, and HSIL was detected in 0.4% of both specimen types. hrHPV positivity was 14.8% in self- and 12.9% in clinician-collected specimens. *Ureaplasma* spp. were frequently detected, and *Candida albicans* was identified in approximately 5% of specimens. Overall agreement was 91.7% (κ = 0.67) for LBC, 95.4% (κ = 0.79) for hrHPV, and 97.0% (κ = 0.72) for STI. **Conclusions:** Integrated cervical self-sampling using a single-step device demonstrated high feasibility and substantial agreement with clinician-based sampling, supporting its potential to improve screening efficiency and reduce participation barriers.

## 1. Introduction

Cervical cancer remains a major public health burden worldwide despite the availability of effective screening strategies and vaccination programs [1,2]. Disparities in screening coverage persist, particularly among underscreened populations and in low- and middle-income countries, where access to healthcare infrastructure is limited [3,4]. High-risk human papillomavirus (hrHPV) testing is now recommended by the World Health Organization as the primary screening method for women aged ≥30 years and older [4]. This shift has accelerated the interest in self-sampling as an alternative to clinician-based specimen collection. Previous studies have shown that self-collected samples provide diagnostic performance comparable to that of clinician-collected samples for hrHPV detection [5,6,7,8]. Self-sampling also offers practical advantages, including improved privacy, convenience, and participation rates [9]. The recent implementation guidance has emphasized HPV self-sampling as an additional strategy to improve screening coverage, particularly among women who are underscreened or face barriers to clinic-based pelvic examination. Accumulating evidence, including systematic reviews and meta-analyses, indicates that HPV testing using self-collected specimens, particularly when PCR-based assays are used, can show diagnostic performance comparable to clinician-collected specimens [10,11].

However, most self-sampling studies have exclusively focused on hrHPV testing. Cytology remains clinically relevant because of its higher specificity in younger women and ability to detect non-HPV-related abnormalities [12,13,14]. The adequacy of cytological specimens depends on the collection of sufficient squamous and glandular/transformation zone components, which may be difficult to achieve with conventional vaginal self-sampling devices [2,15,16,17,18]. Therefore, evaluation of self-sampling devices intended to obtain cervical cellular material should include not only hrHPV detection but also liquid-based cytology adequacy and diagnostic agreement with clinician-collected samples. In addition, sexually transmitted infections (STIs) frequently coexist with HPV infections and contribute to reproductive health morbidity. Molecular testing for sexually transmitted infection-related organisms is another potential application of self-collected genital specimens [19]. Integrating cytology and STI testing into a single self-collected specimen could streamline screening workflows and reduce the need for repeated clinical visits [20,21]. Despite these potential advantages, data evaluating the combined use of self-sampling for cytology, HPV, and STI detection remain limited. Moreover, some previous studies have used vaginal rather than cervical specimens, which may compromise cytological adequacy [22].

The present study aimed to evaluate the feasibility and diagnostic agreement of a novel self-sampling device (Earlypap^®^) that enables simultaneous cytology, HPV, and STI testing using a single sampling procedure. All samples were analyzed for cytology, hrHPV, and STI.

## 2. Materials and Methods

### 2.1. Study Design and Population

This study prospective paired study enrolled 520 women who visited a gynecological clinic (Motaean Woman Hospital, Cheong-ju, Republic of Korea) between December 2024 and February 2025. Inclusion criteria targeted women for routine screening, excluding those with menstruation or a history of cervical surgery. Participant underwent paired self- and clinician-collected cervical sampling. All samples were analyzed for cytology, HPV testing, and STI detection (Supplementary Figure S1). Following the sampling, participants completed a survey evaluating their knowledge of HPV, STI and self-sampling, as well as the perceived ease of use, preferences, and future willingness to utilize in the self-sampling method. The sample size was determined by the number of eligible women who consented to participate during the study period. The study protocol was reviewed and approved by the Institutional Review Board of Chungbuk National University Hospital (IRB No. CBNUH 2024-09-012) and was conducted in accordance with the Declaration of Helsinki. Written informed consent was obtained from all participants.

### 2.2. Sample Collection

All participants underwent paired cervical sampling, consisting of self-sampling using the Earlypap^®^ device followed by clinician-collected sampling using a colposcopic cytobrush. Thus, the women who used the Earlypap^®^ device were the same participants who subsequently underwent clinician-collected cytobrush sampling. Participants first performed self-sampling in a private room using the Earlypap^®^ device (Biodyne, Seoul, Republic of Korea), which consists of a flexible insertion shaft and a collection brush designed to facilitate cervical sampling without the use of a vaginal speculum. Participants were instructed to insert the device until resistance was felt and then rotate the brush five times to collect cellular material. The procedure was standardized using written and visual instructions provided to all participants (Supplementary Figure S3). Subsequently, clinicians collected paired cervical specimens using a colposcopic cytobrush. Each self-collected and clinician-collected sample was placed into a separate 20 mL vial of preservative solution (Biodyne, Seoul, Republic of Korea). Each sample was divided into two aliquots: 7 mL for liquid-based cytology and 7 mL for molecular studies, including HPV and STI detection.

### 2.3. Liquid-Based Cytology

The samples were processed using a PATHPLORER AUTO LBC system (Blowing Technology^TM^, Biodyne, Seoul, Republic of Korea) [13,23,24]. After cell filtration and automated deposition onto glass slides, the prepared slides were fixed in 95% ethanol and stained using a standardized Papanicolaou method. Briefly, the protocol included nuclear staining with Harris’ hematoxylin (Muto Pure Chemicals Co., Ltd., Tokyo, Japan), followed by cytoplasmic counterstaining using Orange G (OG-6) and Eosin Azure (EA-50) solutions (Muto Pure Chemicals). A pathologist (Woo CG) who was blinded to the sampling method independently interpreted the slides in a blinded manner. Cytological results were classified according to the 2014 Bethesda System [25]. The absence of an endocervical/transformation zone component (EC/TZ) was not considered a criterion for an unsatisfactory specimen and was recorded separately.

### 2.4. Molecular Studies for HPV and STI Testing

Genotyping for hrHPV was conducted using the cobas^®^ 4800 system (Roche, Santa Clara, CA, USA), which was designed to provide individual results for HPV 16 and 18, along with a simultaneous, pooled result for the other 12 high-risk genotypes, including subtypes 31, 33, 35, 39, 45, 51, 52, 56, 58, 59, 66, and 68. The assay was performed according to the manufacturer’s protocol, involving automated nucleic acid extraction followed by real-time PCR amplification and target detection. For STI testing (participants aged < 60 years), multiplex real-time PCR was performed using the NextGene™ STI-12 Detection Kit (EONEBIOTECH, Incheon, Republic of Korea), targeting *Neisseria gonorrhoeae*, *Mycoplasma genitalium*, Herpes Simplex Virus (HSV)1, HSV2, *Treponema pallidum*, *Chlamydia trachomatis*, *Mycoplasma hominis*, *Candida albicans*, *Trichomonas vaginalis*, *Ureaplasma parvum*, and *Ureaplasma urealyticum*. Briefly, nucleic acids were extracted from the preserved sample aliquots and amplified using kit-provided primers, probes, PCR master mix, and controls according to the manufacturer’s protocol.

### 2.5. Statistical Analyses

There was no post-enrollment attrition, as all 520 enrolled participants completed both self-collected and clinician-collected cervical sampling. For cytology, hrHPV, and STI testing, agreement between self-collected and clinician-collected samples was assessed using percentage agreement and Cohen’s kappa coefficients (κ), with 95% confidence intervals (CIs). STI analyses were restricted to 504 participants aged < 60 years. Participants who initially failed self-sampling repeated the procedure and were included in the final paired analyses. Age-stratified analyses were conducted as exploratory descriptive analyses to assess age-specific patterns. Analyses were performed using R software (R Foundation, Vienna, Austria) (version 4.5.0).

## 3. Results

### 3.1. Participant Characteristics and Acceptability of Self-Sampling

The largest age group was 30–39 years (44.2%, 230/520), followed by 40–49 years (21.3%, 111/520) and <30 years (20.4%, 106/520) (Supplementary Table S1). Overall, 12.5% (65/520) of the participants reported no prior cervical cancer screening, with the highest proportion observed among women younger than 30 years (33.0%).

Self-sampling was successfully completed on the first attempt by 98.5% (511/520) of the participants. Only nine participants (1.7%, 9/520) required a second attempt because of device contamination or preservative spillage. Most participants rated the procedure as easy to perform (97.3%, 506/520). Preference for self-sampling over clinician collection was reported by 92.1% (479/520) of participants, and 94.6% (492/520) indicated a willingness to use self-sampling in future screening. The median self-sampling time was 100 s (range, 45–282 s), with longer durations observed among participants aged ≥ 60 years (Supplementary Table S1).

### 3.2. Cytology

**Figure 1:**
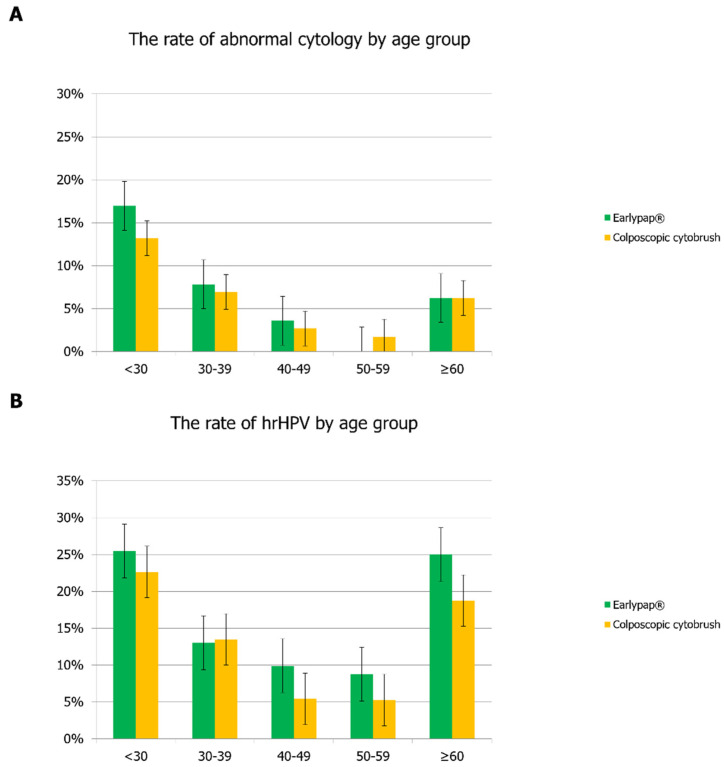
Age-specific distribution of abnormal cytology (**A**) and high-risk human papillomavirus (hrHPV) positivity (**B**) among self-collected and clinician-collected specimens. The x-axis represents age in years, and the y-axis represents prevalence. Error bars represent the standard errors of the proportion.

The unsatisfactory liquid-based cytology rates were comparable between self-collected and clinician-collected specimens (1.4%, 7/520 vs. 1.7%, 9/520, respectively) (Table 1). EC/TZ was identified in 83.1% (432/520) of self-collected specimens and 85.2% (443/520) of clinician-collected specimens. Abnormal cytological findings were detected in both sampling methods, including ASC-US (5.2%, 27/520 in both methods), LSIL (2.3%, 12/520 in self-sampling vs. 1.2%, 6/520 in clinician-sampling), and HSIL (0.4%, 2/520 in both methods). The two women with HSIL identified by self-sampling corresponded to the same individuals diagnosed by clinician-sampling. The overall agreement rate for cytological diagnosis between self-collected and clinician-collected specimens was 91.7% (477/520), with Cohen’s kappa value of 0.67 (95% CI: 0.53–0.82), indicating substantial agreement (Table 1). Age-stratified analysis demonstrated the highest prevalence of abnormal cytology among women younger than 30 years, with decreasing rates in the middle-aged groups and a modest increase among women aged ≥60 years (Supplementary Table S2, Figure 1A).

*Candida albicans* was the only microorganism identified by cytological examination and was detected in 5.6% of the self-collected samples and 5.2% of the clinician-collected samples.

### 3.3. High-Risk Human Papillomavirus Detection

hrHPV was detected in 14.8% (77/520) of the self-collected specimens and 12.9% (67/520) of the clinician-collected specimens (Table 2). Agreement between sampling methods for hrHPV detection was 95.4% (496/520), with a kappa coefficient of 0.79 (95% CI: 0.71–0.87), indicating substantial agreement.

Among hrHPV-positive cases, 17 were detected exclusively in self-collected samples and seven exclusively in clinician-collected samples. Age-stratified analysis showed the highest hrHPV prevalence among women younger than 30 years, followed by a gradual decline in middle-aged groups and a secondary increase among women aged ≥ 60 years (Supplementary Table S3, Figure 1B).

### 3.4. Sexually Transmitted Infection Detection

STI pathogens were identified in 46.8% (236/504) of the self-collected samples and in 44.8% (226/504) of the clinician-collected samples. The overall agreement rate was 97.0% (487/504), with a kappa coefficient of 0.72 (95% CI: 0.65–0.78), indicating substantial agreement (Table 3). Ureaplasma species were the most frequently detected organisms in both sampling methods, accounting for approximately 40% of positive cases. *Candida albicans* was detected in approximately 5% of the samples. Chlamydia trachomatis was identified in three self-collected and two clinician-collected samples. STI prevalence was the highest among participants younger than 30 years and decreased progressively with age.

### 3.5. Cervical Biopsy Outcomes Following Screening

Colposcopy and biopsy were performed selectively as a routine clinical care, based on screening results, HPV genotype, colposcopic fin dings, previous screening history, patient preference, and the judgment of the gynecologist. Biopsy was not mandated by the study protocol for all participants with hrHPV positivity or abnormal cytology. In addition, some participants were lost to follow-up and some declined colposcopy or biopsy. Among women who were hrHPV-positive but cytology-negative, two (2.6% of 77 self-samples and 3.0% of 67 clinician-samples) underwent biopsy (Supplementary Table S4). These two individuals were the same across both collection methods. The biopsy results were negative in one case and HSIL in one case. Among the 19 women who tested positive for ASC-US and hrHPV in self-sampling, eight (42.1%, 8/19) underwent biopsy, resulting in histological confirmation of LSIL in six cases and HSIL in two cases. Five participants (41.7%, 5/12) with LSIL and hrHPV positivity in self-sampling underwent biopsy, all of whom were diagnosed with LSIL. Two participants with HSIL and hrHPV positivity identified by self-sampling and clinician sampling were confirmed to have HSIL on histopathological examination.

## 4. Discussion

Our findings are broadly consistent with recent published evidence on self-collected specimens for cervical cancer screening. The meta-analysis of agreement between self-collected and clinician-collected samples for HPV testing reported a pooled overall agreement of 88.7% and a pooled kappa value of 0.72 [26]. In the present study, hrHPV testing showed a higher overall agreement of 95.4% and a kappa coefficient of 0.79, supporting the concordance of self-collected specimens with clinician-collected specimens when a PCR-based HPV assay is used. Another study reported that HPV testing using self-collected specimens showed comparable clinical accuracy to clinician-collected HPV testing and higher sensitivity than clinician-collected cytology [10]. These findings support the clinical relevance of HPV self-sampling, while also emphasizing that HPV self-collection is primarily a molecular screening approach. A recent systematic review and meta-analysis compared patient-collected and clinician-collected cervical cytology for CIN screening [27]. In the present study, self-collected specimens showed a low unsatisfactory rate and substantial agreement with clinician-collected cytology (κ = 0.67), including concordant detection of both HSIL cases. These findings suggest that the Earlypap^®^ device may provide cervical cellular material suitable for liquid-based cytology. However, our cytology results should be interpreted as preliminary and require validation in larger cohorts with systematic histological follow-up.

This study demonstrated that cervical self-sampling using the Earlypap^®^ device provides feasibility and substantial diagnostic agreement with clinician-collected specimens across LBC, hrHPV, and STI detection. Importantly, the novelty of this study lies not in the individual detection of hrHPV or STIs using self-collected samples established in previous studies, but in the integration of cytology, hrHPV testing, and STI detection from a single self-collected cervical specimen. The first-attempt success rate (98.5%, 511/520) and participant preference for self-sampling (92.1%, 479/520) indicate that this approach is both practical and acceptable in routine screening settings. Unlike many self-sampling approaches based primarily on vaginal specimens for molecular testing, the present device was intended for cervical sampling without a speculum (Supplementary Figures S2 and S3). The low unsatisfactory cytology rate and detection of cytologic abnormalities, including HSIL, support the feasibility of obtaining diagnostically adequate cervical material (Supplementary Figure S4).

The collected cells were preserved in liquid medium that maintained cellular morphology for LBC, while preserving nucleic acids for multiplex PCR, thereby enabling three tests from a single collection. Although ThinPrep is a widely recognized technology, the PATHPLORER LBC system used in this study also provides high-quality cellular visualization and has demonstrated substantial agreement with histological outcomes in previous studies [12,18]. The low rate of unsatisfactory slides in our self-sampling group further confirms that the combination of the Earlypap^®^ device and LBC system is robust enough for routine clinical use.

Consistent with previous reports, self-collected specimens showed substantial agreement with clinician-collected samples for hrHPV detection (κ = 0.79) [7,28,29,30]. Similarly, STI detection showed substantial concordance (κ = 0.72), in line with previous studies using self-sampling [31]. Our findings extend the existing evidence by demonstrating that a single self-collected cervical specimen can be used for multi-modality testing, including cytological evaluation (κ = 0.67), which is not routinely feasible with most self-sampling devices. This integrated approach may reduce the need for multiple sampling procedures and improve the efficiency of cervical cancer screening programs.

Molecular testing for SIT revealed a high detection frequency of *Ureaplasma* spp. (43.3% vs. 41.3%) and *Mycoplasma hominis* (5.6% vs. 4.6%) in both self-collected and clinician-collected specimens. *Candida albicans* was detected in approximately 5% of specimens. A clinical distinction should be made regarding the organisms included in STI detection. Rout routine screening and treatment for *Mycoplasma* and *Ureaplasma* spp. are not recommended in asymptomatic women. In addition, *Candida albicans* is not classified as a STI, although it is detected in vulvovaginal candidiasis. Therefore, the detection of these organisms should not be interpreted as evidence supporting broad routine STI screening or treatment in asymptomatic populations. Clinical implementation should be guided by established screening and treatment recommendations. In this context, the potential clinical value of self-collected molecular testing may be greatest when applied to guideline-supported STI targets, such as Chlamydia trachomatis, Neisseria gonorrhoeae, and Trichomonas vaginalis, particularly in populations with barriers to clinic-based testing.

Age-stratified analyses revealed the highest prevalence of abnormal cytology, hrHPV, and STIs among younger participants, with declining rates in middle-aged groups and a secondary increase among women aged ≥ 60 years. These age-specific patterns suggest that self-sampling may have potential utility across different age groups, although the present study did not directly assess the barriers to clinic-based screening. Self-sampling offers several advantages from a public health perspective. It reduces the logistical barriers associated with clinic visits, minimizes discomfort and privacy concerns, and may increase screening participation among underscreened populations. An integrated approach to perform cytology, HPV testing, and STI detection from a single specimen may help streamline screening pathways and reduce repeat sampling or additional clinic visits.

This study has some limitations. First, histological evaluation was not performed in all participants with abnormal screening results, and biopsy was obtained only in selected patients as part of routine clinical management. In real-world practice, some participants were lost to follow-up and some declined colposcopy or biopsy. Therefore, the biopsy outcomes in this study provide limited follow-up information and should not be regarded as a histological reference standard for evaluating the diagnostic accuracy. Second, the study was conducted at a single clinical site, and participants were recruited from a clinic-based screening population, potentially limiting its generalizability to other populations and healthcare systems. Third, the sample size may be insufficient to establish definitive equivalence between self-collected and clinician-collected samples, and to represent a broad population. Fourth, cytological evaluation was performed using a single LBC system, and the results may not be directly generalizable to other validated technologies, such as ThinPrep and SurePath. These findings should be interpreted as preliminary evidence of feasibility and agreement rather than conclusive validation of diagnostic performance. Future large-scale studies incorporating multiple LBC platforms are required to validate this integrated self-sampling method. Finally, an important limitation is the requirement for laboratory infrastructure. Although self-sampling may reduce patient-level barriers, integrated testing from a single specimen still requires appropriate laboratory systems. Access to such laboratory facilities, trained personnel, and specimen transport systems may be limited in rural or resource-limited settings, where self-sampling could otherwise have substantial public health value. Therefore, implementation of this approach would require consideration of local laboratory capacity, referral networks, and specimen logistics.

## 5. Conclusions

Single-step cervical self-sampling using Earlypap^®^ is a reliable alternative to clinician-based sampling, enabling integrated cytology, HPV, and STI testing from a single specimen.

## Figures and Tables

**Table 1:** Comparison of liquid-based cytology between Earlypap^®^ self-collected specimens and clinician-collected colposcopic cytobrush specimens.

Cytology		Colposcopic Cytobrush: *n* (%)						Concordance	Kappa Coefficient (95% CI)
		UE ^1^	NILM ^2^	ASC-US ^3^	LSIL ^4^	HSIL ^5^	Total		
Earlypap^®^	UE	1 (0.2)	6 (1.2)	0 (0.0)	0 (0.0)	0 (0.0)	7 (1.4)	477 (91.7)	0.67 (0.53–0.82)
	NILM	8 (1.5)	456 (87.7)	7 (1.3)	1 (0.2)	0 (0.0)	472 (90.8)		
	ASC-US	0 (0.0)	9 (1.7)	18 (3.5)	0 (0.0)	0 (0.0)	27 (5.2)		
	LSIL	0 (0.0)	5 (1.0)	2 (0.4)	5 (1.0)	0 (0.0)	12 (2.3)		
	HSIL	0 (0.0)	0 (0.0)	0 (0.0)	0 (0.0)	2 (0.4)	2 (0.4)		
	Total	9 (1.7)	476 (91.5)	27 (5.2)	6 (1.2)	2 (0.4)	520 (100)		

^1^ UE, Unsatisfactory for evaluation; ^2^ NILM, Negative for intraepithelial lesion or malignancy; ^3^ ASC-US, Atypical squamous cells of undetermined significance; ^4^ LSIL, Low grade squamous intraepithelial lesion; ^5^ HSIL, High grade squamous intraepithelial lesion.

**Table 2:** Agreement of high-risk human papillomavirus between Earlypap^®^ self-collected specimens and clinician-collected colposcopic cytobrush specimens.

HPV Status		Coloposcopic Cytobrush (Clinician): *n* (%)					Concordance	Kappa Coefficient (95% CI)
		Negative	HPV 16	HPV 18	Other hrHPV	Total		
Earlypap^®^ (self)	Negative	436 (83.8)	0 (0.0)	1 (0.2)	6 (1.2)	443 (85.2)	496 (95.4)	0.79 (0.71–0.87)
	HPV 16	0 (0.0)	8 (1.5)	0 (0.0)	1 (0.2)	9 (1.7)		
	HPV 18	0 (0.0)	0 (0.0)	1 (0.2)	0 (0.0)	1 (0.2)		
	other hrHPV	17 (3.3)	0 (0.0)	0 (0.0)	51 (9.8)	68 (13.1)		
	Total	453 (87.1)	8 (1.5)	2 (0.4)	58 (11.2)	520 (100)		

**Table 3:** Detection of sexually transmitted infection-related and genital tract microorganisms according to sampling method and age group.

Age (yrs)	Earlypap^®^ (Self): *n* (%)							Coloposcopic Cytobrush (Clinician): *n* (%)							Kappa Coefficient (95% CI)
	Negative	UU/UP ^1^	MG ^2^	MH ^3^	HSV2 ^4^	Candida ^5^	CT ^6^	Negative	UU/UP	MG	MH	HSV2	Candida	CT	
Total	268 (53.2)	218 (43.3)	4 (0.8)	28 (5.6)	4 (0.8)	28 (5.6)	3 (0.6)	278 (55.2)	208 (41.3)	3 (0.6)	23 (4.6)	6 (1.2)	26 (5.2)	2 (0.4)	0.72 (95% CI: 0.65–0.78)
<30	48 (45.3)	55 (51.9)	2 (1.9)	7 (6.6)	0 (0.0)	7 (6.6)	1 (0.9)	48 (45.3)	55 (51.9)	3 (2.8)	7 (6.6)	0 (0.0)	7 (6.6)	1 (0.9)	
30–39	119 (51.7)	100 (43.5)	2 (0.9)	11 (4.8)	3 (1.3)	14 (6.1)	1 (0.4)	128 (55.7)	93 (40.4)	0 (0.0)	6 (2.6)	3 (1.3)	13 (5.7)	1 (0.4)	
40–49	66 (59.5)	43 (38.7)	0 (0.0)	5 (4.5)	1 (0.9)	3 (2.7)	0 (0.0)	66 (59.5)	42 (37.8)	0 (0.0)	5 (4.5)	2 (1.8)	3 (2.7)	0 (0.0)	
50–59	35 (61.4)	20 (35.1)	0 (0.0)	5 (8.8)	0 (0.0)	4 (7.0)	1 (1.8)	36 (63.2)	18 (31.6)	0 (0.0)	5 (8.8)	1 (1.8)	3 (5.3)	0 (0.0)	

^1^ UU/UP, Ureaplasma urealyticum and/or Ureaplasma parvum; ^2^ MG, Mycoplasma genitalium; ^3^ MH, Mycoplasma hominis; ^4^ HSV2, Herpes simplex virus 2; ^5^ Candida, Candida albicans; ^6^ CT, Chlamydia trachomatis.

## Data Availability

The datasets generated and analyzed during the current study are available from the corresponding author upon reasonable request.

## Supplementary Materials

The following supporting information can be downloaded at https://www.mdpi.com/article/10.3390/diagnostics16121863/s1: Figure S1: Study design; Figure S2: Earlypap^®^ self-sampling device; Figure S3: Standardized written and visual self-sampling instructions; Figure S4: Representative liquid-based cytology findings including low-grade squamous intraepithelial lesion (A), high-grade squamous intraepithelial lesion (B), and Candida albicans (C); Table S1: Participants’ characteristics; Table S2: Cytological diagnosis using Earlypap^®^ and colposcopic cytobrush by age group; Table S3: High risk HPV prevalence using Earlypap^®^ and colposcopic cytobrush by age groups; Table S4: Cervical biopsy after cervical cancer screening.

## Abbreviations

The following abbreviations are used in this manuscript:

hrHPV	high-risk human papillomavirus
LBC	liquid-based cytology
EC/TZ	endocervical/transformation zone component
STI	sexually transmitted infection
CI	confidence interval
