# Supplementary material for: Integrated Cervical Self-Sampling for Cytology, High-Risk Human Papillomavirus, and Sexually Transmitted Infection Testing: A Prospective Study
Source: Diagnostics (Basel). 2026 Jun 16;16(12):1863. doi: 10.3390/diagnostics16121863 (PMC13297677; doi:10.3390/diagnostics16121863)

Figure S1

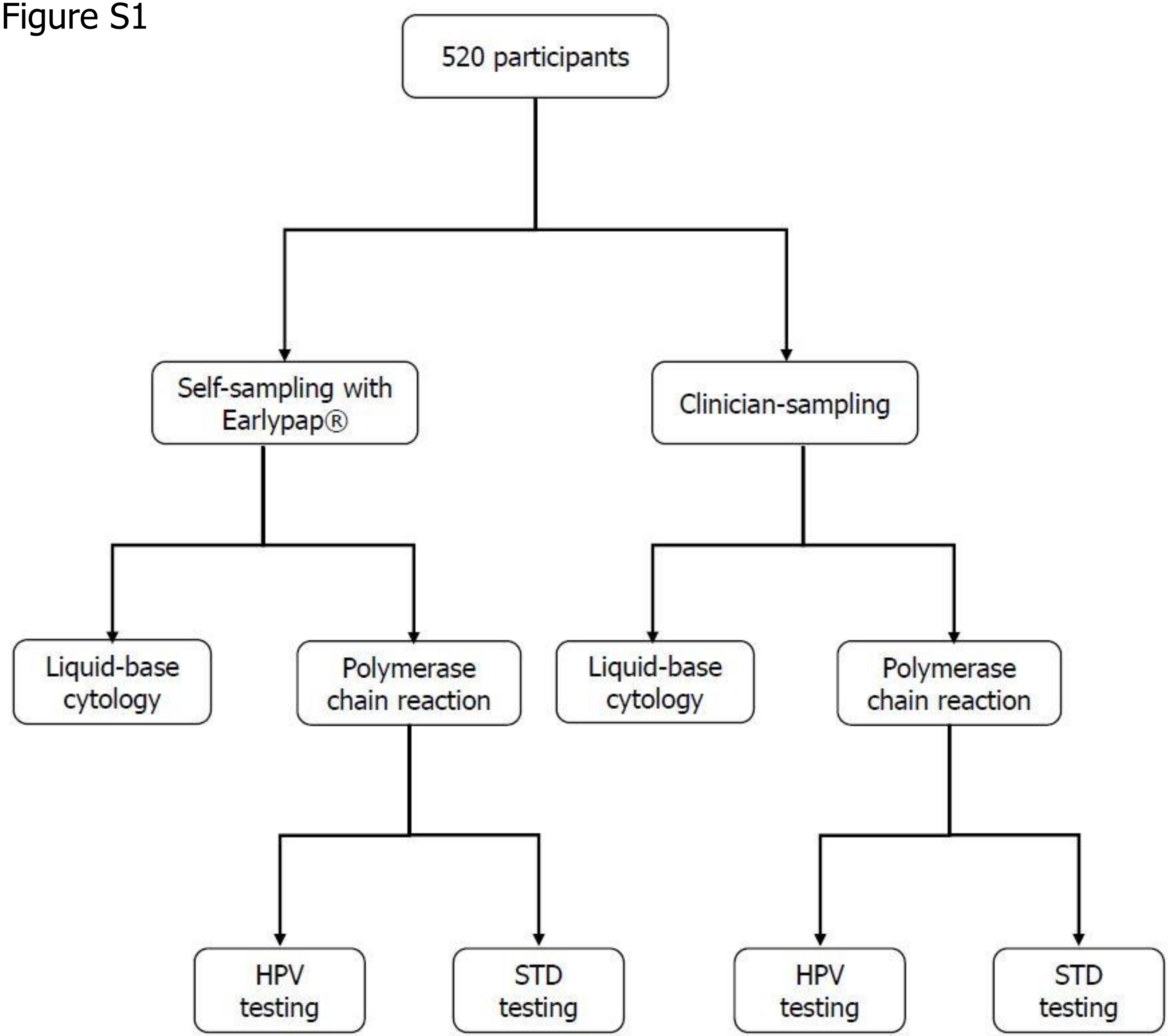

520 participants
Self-sampling with Earlypap®
Clinician-sampling
Liquid-base cytology
Polymerase chain reaction
Liquid-base cytology
Polymerase chain reaction
HPV testing
STD testing
HPV testing
STD testing

Figure S2

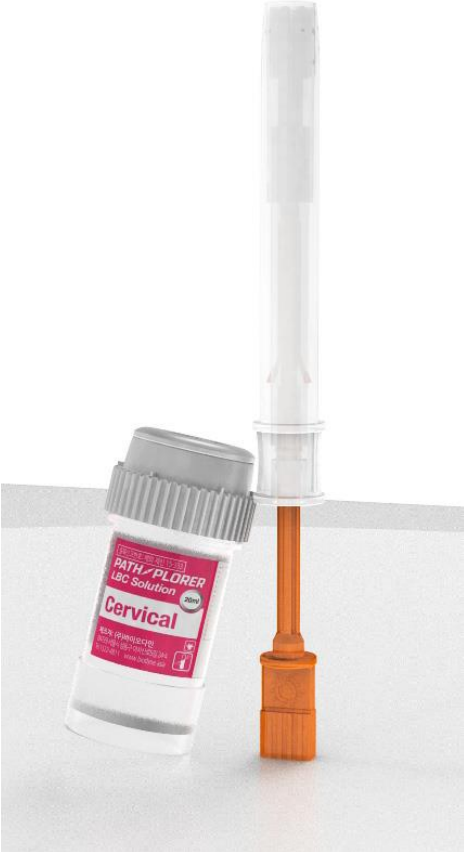

Figure S3

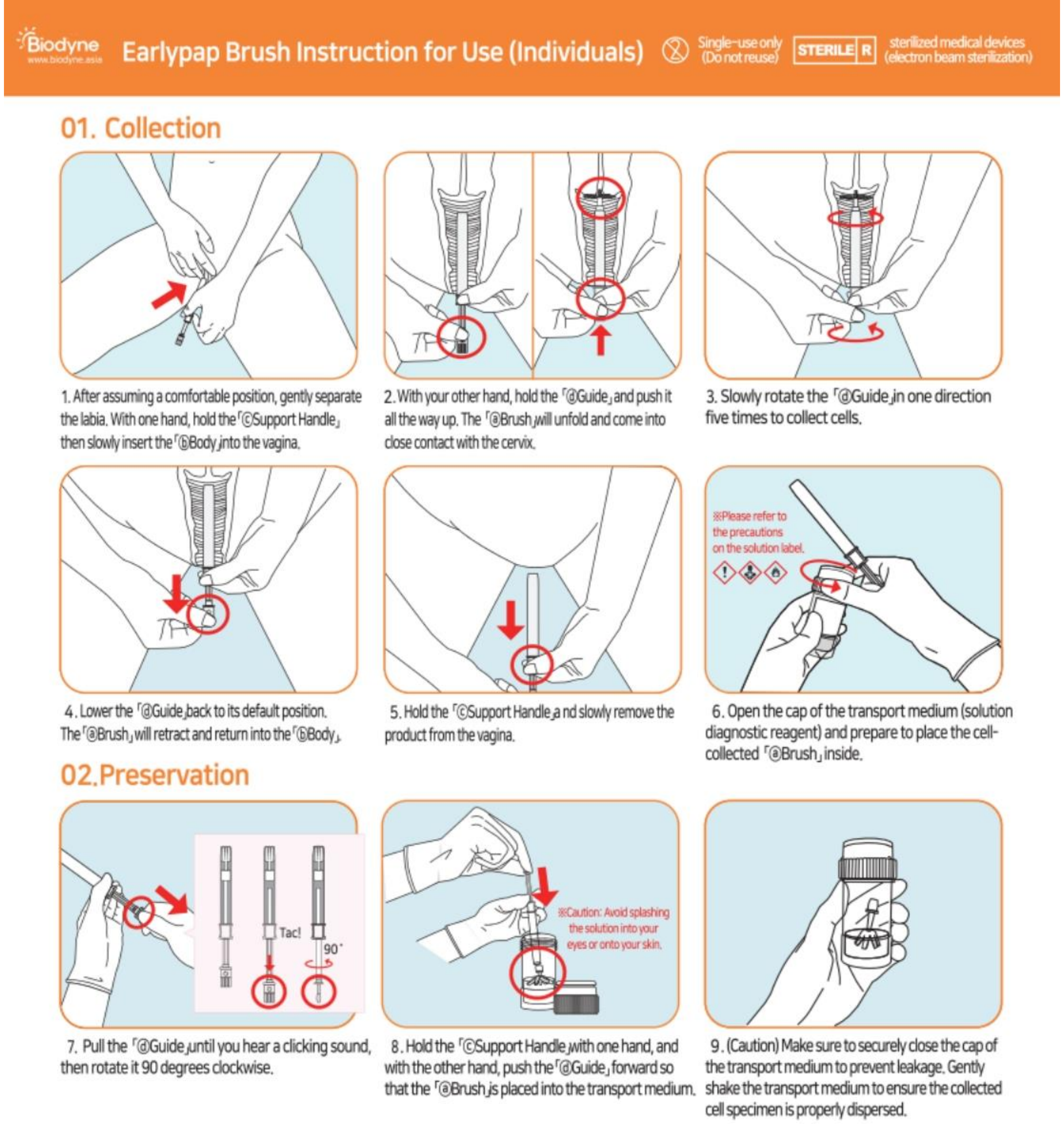
Biodyne
www.biodyne.asia
Earlypap Brush Instruction for Use (Individuals)
Single-use only (Do not reuse)
STERILE R
sterilized medical devices (electron beam sterilization)
01. Collection
1. After assuming a comfortable position, gently separate the labia. With one hand, hold the「ⓒSupport Handle」 then slowly insert the「ⓑBody」into the vagina.
2. With your other hand, hold the「ⓐGuide」and push it all the way up. The「ⓐBrush」will unfold and come into close contact with the cervix.
3. Slowly rotate the「ⓐGuide」in one direction five times to collect cells.
4. Lower the「ⓐGuide」back to its default position. The「ⓐBrush」will retract and return into the「ⓑBody」.
5. Hold the「ⓒSupport Handle」a nd slowly remove the product from the vagina.
※Please refer to the precautions on the solution label.
6. Open the cap of the transport medium (solution diagnostic reagent) and prepare to place the cell-collected「ⓐBrush」inside.
02.Preservation
Tac!
90˚
7. Pull the「ⓐGuide」until you hear a clicking sound, then rotate it 90 degrees clockwise.
※Caution: Avoid splashing the solution into your eyes or onto your skin.
8. Hold the「ⓒSupport Handle」with one hand, and with the other hand, push the「ⓐGuide」forward so that the「ⓐBrush」is placed into the transport medium.
9. (Caution) Make sure to securely close the cap of the transport medium to prevent leakage. Gently shake the transport medium to ensure the collected cell specimen is properly dispersed.

Figure S4A

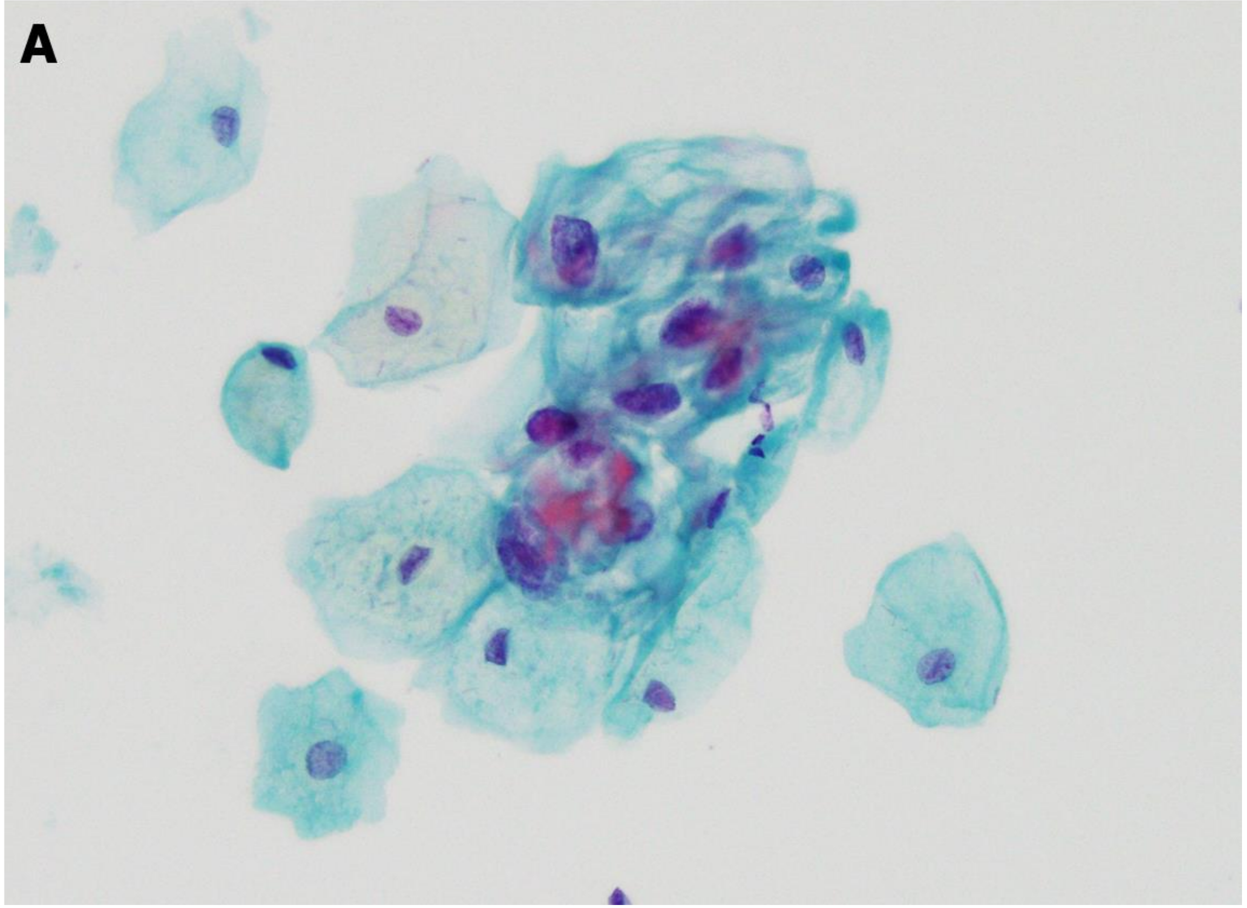

Figure S4B

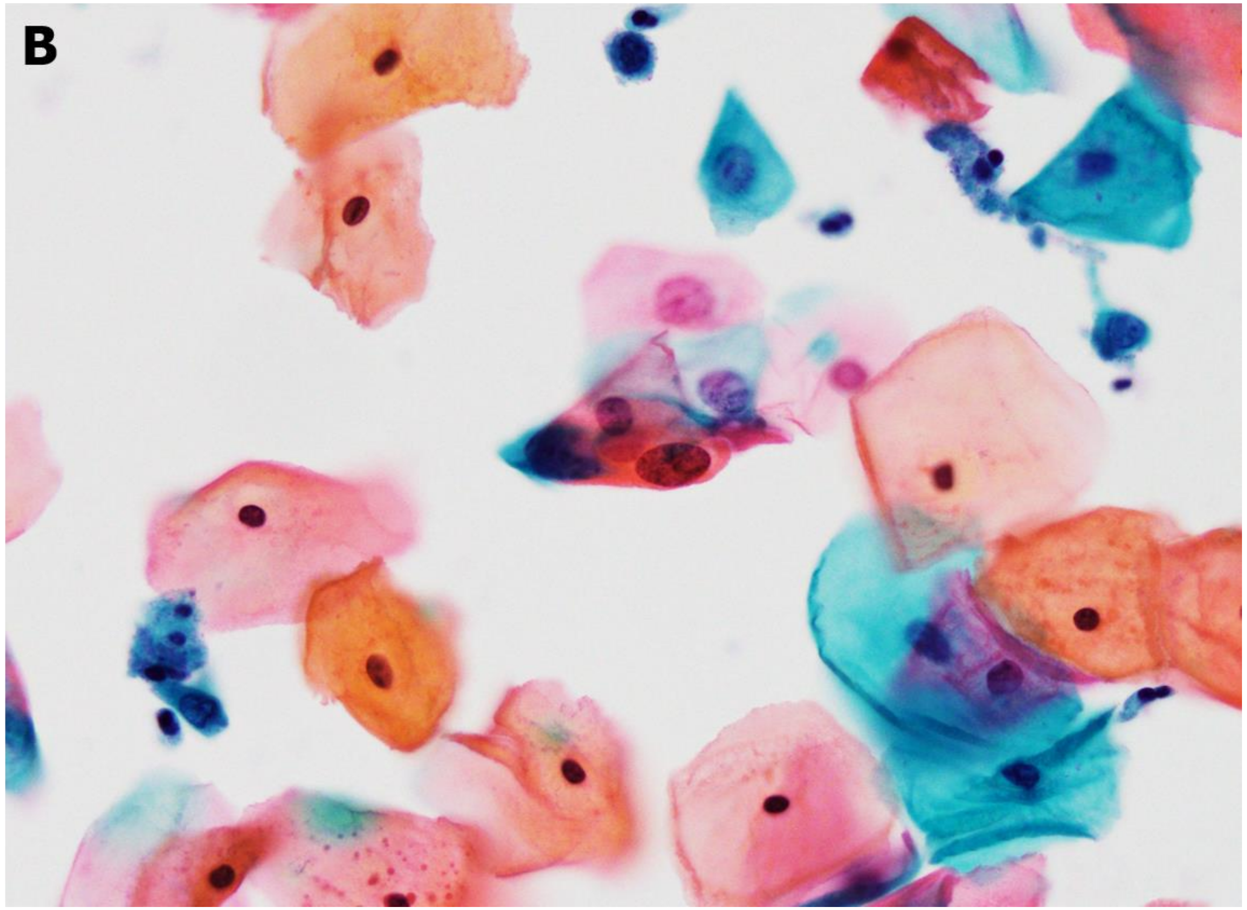

Figure S4C

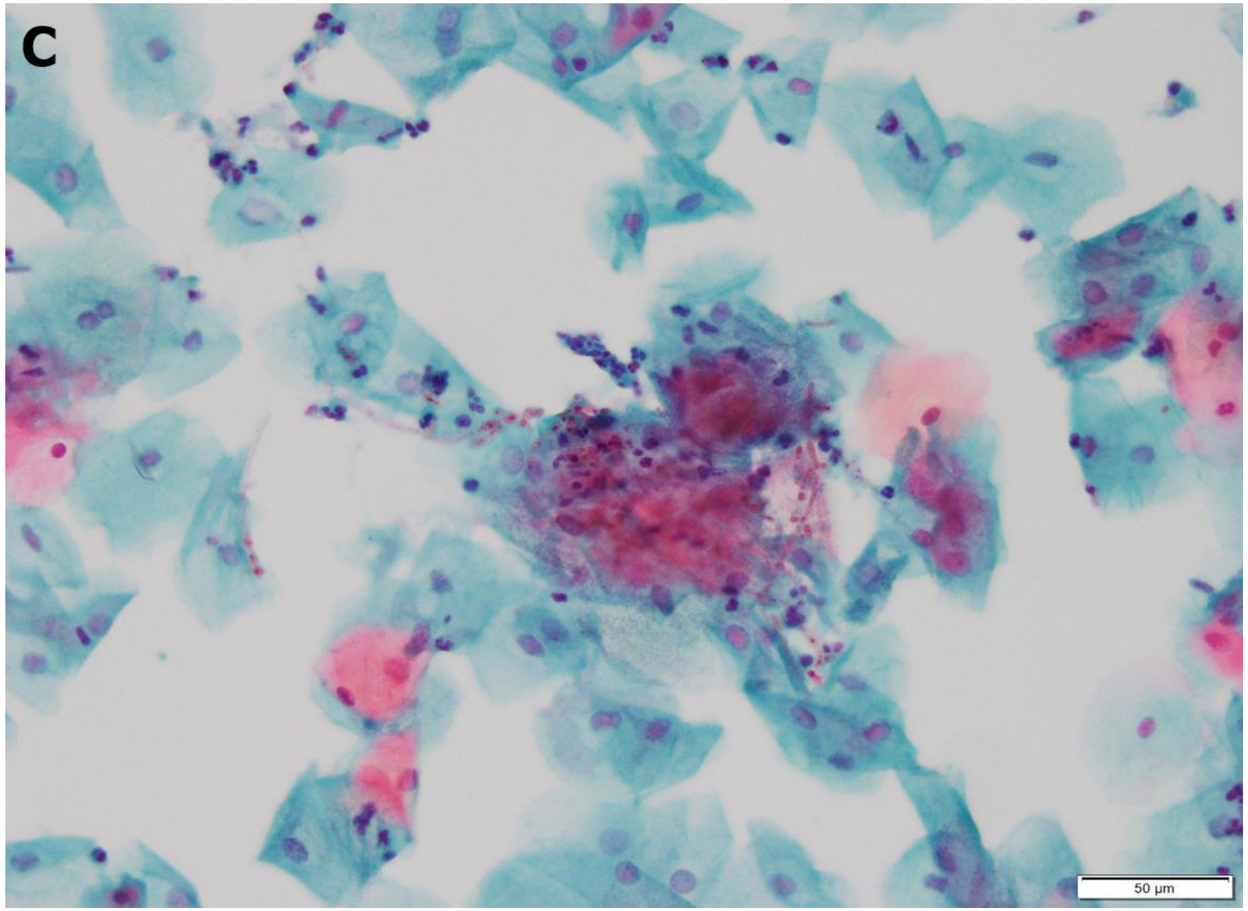

Supplement: Supplementary file 1 [file diagnostics-16-01863-s001.zip › Figures S1–S4.pdf]
