# Supplementary material for: Integrated Cervical Self-Sampling for Cytology, High-Risk Human Papillomavirus, and Sexually Transmitted Infection Testing: A Prospective Study
Source: Diagnostics (Basel). 2026 Jun 16;16(12):1863. doi: 10.3390/diagnostics16121863 (PMC13297677; doi:10.3390/diagnostics16121863)
Supplement: Supplementary file 1 [file diagnostics-16-01863-s001.zip › Tables S1–S4.pdf]

Supplementary Table S1. Participants’ characteristics

Characteristics	Total	Age (yrs)				
		<30	30-39	40-49	50-59	≥60
Participants: n (%)	520 (100)	106 (20.4)	230 (44.2)	111 (21.3)	57 (11.0)	16 (3.1)
History of screening						
Never	65 (12.5)	35 (33.0)	17 (7.4)	7 (6.3)	4 (7.0)	2 (12.5)
Screened	455 (87.5)	71 (67.0)	213 (92.6)	104 (93.7)	53 (93.0)	14 (87.5)
Visits to a gynecologist more than twice per year						
Yes	110 (21.2)	33 (31.1)	46 (20.0)	20 (18.0)	11 (19.3)	0 (0.0)
No	410 (78.8)	73 (68.9)	184 (80.0)	91 (82.0)	46 (80.7)	16 (100)
Heard of HPV testing						
Yes	339 (65.2)	76 (71.7)	167 (72.6)	69 (62.2)	22 (38.6)	5 (31.3)
No	181 (34.8)	30 (28.3)	63 (27.4)	42 (37.8)	35 (61.4)	11 (68.8)
Heard of STI testing						
Yes	291 (56.0)	75 (70.8)	132 (57.4)	52 (46.8)	28 (49.1)	4 (25.0)
No	229 (44.0)	31 (29.2)	98 (42.6)	59 (53.2)	29 (50.9)	12 (75.0)
Heard of self-sampling						
Yes	14 (2.7)	4 (3.8)	6 (2.6)	2 (1.8)	1 (1.8)	1 (6.3)
No	506 (97.3)	102 (96.2)	224 (97.4)	109 (98.2)	56 (98.2)	15 (93.8)
Ease of self-sampling						
Easy	506 (97.3)	103 (97.2)	227 (98.7)	109 (98.2)	53 (93.0)	14 (87.5)
Difficult	14 (2.7)	3 (2.8)	3 (1.3)	2 (1.8)	4 (7.0)	2 (12.5)
Self-sampling on the first attempt						
Success	511 (98.5)	104 (98.1)	227 (98.7)	110 (99.1)	55 (96.5)	15 (93.8)
Failure	9 (1.7)	2 (1.9)	3 (1.3)	1 (0.9)	2 (3.5)	1 (6.3)
Reliability of self-sampling						
Yes	481 (92.5)	97 (91.5)	207 (90.0)	106 (95.5)	56 (98.2)	15 (93.8)
No	39 (7.5)	9 (8.5)	23 (10.0)	5 (4.5)	1 (1.8)	1 (6.3)
Preference of sampling						
Self	479 (92.1)	102 (96.2)	210 (91.3)	103 (92.8)	53 (93.0)	11 (68.8)
Clinician	41 (7.9)	4 (3.8)	20 (8.7)	8 (7.2)	4 (7.0)	5 (31.3)
Willingness of self-sampling						
Yes	492 (94.6)	104 (98.1)	212 (92.2)	108 (97.3)	56 (98.2)	12 (75.0)
No	28 (5.4)	2 (1.9)	18 (7.8)	3 (2.7)	1 (1.8)	4 (25.0)
Willingness to introduce self-sampling to others						
Yes	512 (98.5)	106 (100)	225 (97.8)	109 (98.2)	57 (100)	15 (93.8)
No	8 (1.5)	0 (0.0)	5 (2.2)	2 (1.8)	0 (0.0)	1 (6.3)
Self-sampling duration: median second (range)	100 (45-282)	98 (67-170)	106 (50-282)	87 (45-150)	100 (57-130)	145 (142-149)

Supplementary Table S2. Cytological diagnosis using Earlypap® and colposcopic cytobrush by age group

Age (yrs)	Earlypap® (self): n (%)						Coloposcopic cytobrush (clinician): n (%)					
	UE[1]	NILM[2]	Candida	ASC-US[3]	LSIL[4]	HSIL[5]	UE	NILM	Candida	ASC-US	LSIL	HSIL
Total	7 (1.4)	472 (90.8)	29 (5.6)	**27 (5.2)**	**12 (2.3)**	**2 (0.4)**	9 (1.7)	476 (91.5)	27 (5.2)	**27 (5.2)**	**6 (1.2)**	**2 (0.4)**
<30	1 (0.9)	87 (82.1)	7 (6.6)	**9 (8.5)**	**8 (7.6)**	**1 (0.9)**	3 (2.8)	89 (84.0)	7 (6.6)	**10 (9.4)**	**3 (2.8)**	**1 (0.9)**
30-39	1 (0.4)	211 (91.7)	14 (6.1)	**13 (5.7)**	**4 (1.7)**	**1 (0.4)**	1 (0.4)	213 (92.6)	12 (5.2)	**12 (5.2)**	**3 (1.3)**	**1 (0.4)**
40-49	0 (0.0)	107 (96.4)	5 (4.5)	**4 (3.6)**	0 (0.0)	0 (0.0)	2 (1.8)	106 (95.5)	6 (5.4)	**3 (2.7)**	0 (0.0)	0 (0.0)
50-59	2 (3.5)	55 (96.5)	3 (5.7)	0 (0.0)	0 (0.0)	0 (0.0)	0 (0.0)	56 (98.3)	2 (3.5)	**1 (1.8)**	0 (0.0)	0 (0.0)
≥60	3 (18.8)	12 (75.0)	0 (0.0)	**1 (6.3)**	0 (0.0)	0 (0.0)	3 (18.8)	12 (75.0)	0 (0.0)	**1 (6.3)**	0 (0.0)	0 (0.0)

1; UE, Unsatisfactory for evaluation
2; NILM, Negative for intraepithelial lesion or malignancy
3; ASC-US, Atypical squamous cells of undetermined significance
4; LSIL, Low grade squamous intraepithelial lesion
5; HSIL, High grade squamous intraepithelial lesion

Supplementary Table S3. High risk HPV prevalence using Earlypap® and colposcopic cytobrush by age groups

Age (yrs)	Earlypap® (self): n (%)				Coloposcopic cytobrush (clinician): n (%)			
	Negative	HPV16	HPV18	other hrHPV	Negative	HPV16	HPV18	other hrHPV
Total (%)	443 (85.2)	**9 (1.7)**	**1 (0.2)**	**68 (13.1)**	453 (87.1)	**8 (1.5)**	**2 (0.4)**	**57 (11.0)**
<30	79 (74.5)	**2 (1.9)**	0 (0.0)	**26 (24.5)**	82 (77.4)	**1 (0.9)**	0 (0.0)	**23 (21.7)**
30-39	200 (87.0)	**4 (1.7)**	0 (0.0)	**26 (11.3)**	199 (86.5)	**4 (1.7)**	**1 (0.4)**	**26 (11.3)**
40-49	100 (90.1)	0 (0.0)	**1 (0.9)**	**10 (9.0)**	105 (94.6)	0 (0.0)	**1 (0.9)**	**5 (4.5)**
50-59	52 (91.2)	**2 (3.5)**	0 (0.0)	**3 (5.3)**	54 (94.8)	**2 (3.5)**	0 (0.0)	**1 (1.8)**
≥60	12 (75.0)	**1 (6.3)**	0 (0.0)	**3 (18.8)**	13 (81.3)	**1 (6.3)**	0 (0.0)	**2 (12.5)**

Supplementary Table S4. Cervical biopsy after cervical cancer screening

The results by sampling	Follow-up loss or no biopsy	Cervical biopsy: n (%)				Total
		Total	Negative	LSIL	HSIL	
EarlyPap® (self-sampling)						
ASC-US	27 (100)	0 (0)				27
hrHPV	75 (97.4)	2 (2.6)	1 (50.0)	0 (0)	1 (50.0)	77
ASC-US and hrHPV	11 (57.9)	8 (42.1)	0 (0)	6 (75.0)	2 (25.0)	19
LSIL and hrHPV	7 (58.3)	5 (41.7)	0 (0)	5 (100)	0 (0)	12
HSIL and hrHPV	0 (0)	2 (100)	0 (0)	0 (0)	2 (100)	2
Colposcopic cytobrush (clinician-sampling)						
ASC-US	27 (100)	0 (0)				27
hrHPV	65 (97.0)	2 (3.0)	1 (50.0)	0 (0)	1 (50.0)	67
ASC-US and hrHPV	12 (60.0)	8 (40.0)	0 (0)	6 (75.0)	2 (25.0)	20
LSIL and hrHPV	3 (50.0)	3 (50.0)	0 (0)	3 (100)	0 (0)	6
HSIL and hrHPV	0 (0)	2 (100)	0 (0)	0 (0)	2 (100)	2
